# The Effect of Feeding Roughages of Varying Digestibility Prepartum on Energy Status and Metabolic Profiles in Beef Cows around Parturition

**DOI:** 10.3390/ani10030496

**Published:** 2020-03-16

**Authors:** Mikaela Jardstedt, Elisabet Nadeau, Mette Olaf Nielsen, Peder Nørgaard, Anna Hessle

**Affiliations:** 1 Department of Animal Environment and Health, Swedish University of Agricultural Sciences, Box 234, 532 23 Skara, Sweden; elisabet.nadeau@slu.se (E.N.);; 2 The Rural Economy and Agricultural Society Sjuhärad, Box 5007, 514 05 Länghem, Sweden; 3 Department of Animal Science, Aarhus University, Blichers Allé 20, 8830 Tjele, Denmark; mon@anis.au.dk; 4 Department of Veterinary and Animal Sciences, University of Copenhagen, Grønnegårdsvej 3, 1870 Frederiksberg C, Denmark; pen@sund.ku.dk

**Keywords:** beef cow, body condition score, energy status, gestation, metabolic profile, roughage

## Abstract

**Simple Summary:**

Grass silages based on timothy-meadow fescue are commonly fed to pregnant beef cows during winter. As beef cows usually are given free access to roughage for rational reasons, the use of these silages has been questioned due to their relatively high nutritional value, which may result in nutrient intakes above animal requirements and, hence, a waste of resources. Therefore, other roughage alternatives are requested, but their effects on cow intake and energy status before calving must be evaluated before applied in practice. Four diets based on timothy-meadow fescue silage, festulolium silage plus urea, reed canarygrass silage or barley straw supplemented with urea and rapeseed meal were fed in free access to mature pregnant beef cows. Timothy-meadow fescue and festulolium diets resulted in overfeeding of energy and protein and in body weight and body condition gains, whereas the opposite was observed for cows fed the other two diets. Hence, reed canarygrass or barley straw supplemented with urea and rapeseed meal prepartum may be suitable alternatives to the traditional timothy-meadow fescue diet, if cows are able to regain lost BCS during the grazing period, and may reduce winter feed costs of the cow-calf producer due to the low intakes of these diets.

**Abstract:**

Resource efficient winter-feeding of mature pregnant beef cows requires knowledge of how different roughage-based feeding strategies affect cow intake and energy status. Four diets based on traditional timothy-meadow fescue silage (TM), festulolium silage plus urea (FE), reed canarygrass silage (RC) or barley straw supplemented with urea and rapeseed meal (BR), were fed ad libitum for 16 weeks prepartum to 36 Hereford cows. Postpartum, cows were fed the same diet before release on pasture. Individual data on cow intake, changes in body weight (BW), body condition score (BCS) and plasma metabolites, calf birth and weaning weights were recorded. The TM and FE diets resulted in increased BW and BCS prepartum (*p* < 0.001), while the RC and BR diets resulted in a catabolic state, as indicated by a loss of BCS, lower insulin levels and higher non-esterified fatty acid levels in cows fed BR (*p* < 0.001). There were no dietary effects on calf parameters (*p* > 0.29). Feeding RC or BR prepartum might be a possible alternative to traditional timothy-meadow fescue silage if cows are allowed to regain lost BCS during the grazing period. The influence on cow reproductive- and calf performance should be considered before making this management change.

## 1. Introduction

Globally, beef cow and calf (suckler-based beef) production is based primarily on different types of grasses, fed either as grazed and/or conserved forage. In the Nordic countries, year-round grazing is not possible due to the climatic conditions. In Sweden, cows are therefore commonly wintered indoors and fed conserved forage during this period. As the cost of harvested grass usually is greater than that of grazing [1], winter feed costs constitute a substantial proportion of the total annual costs of feeding the cow [1,2]. 

Beef cows are usually fed only roughage ad libitum during winter for rational reasons, as this strategy saves time for labor and, hence, reduces cost of production. Furthermore, stables for beef cows are commonly built to provide one eating space per three cows, which require cows to be fed ad libitum according to the Swedish Board of Agriculture regulations and general recommendations (SJVFS 2012:26). In addition, one third of the Swedish beef cows are managed according to the organic rules of production, which require that cows have ad libitum access to roughage. Most beef cows calve in spring and, hence, their nutrient demands are relatively low during a large part of the winter [3]. In order to constrain feed intake of spring-calving mature beef cows at ad libitum feeding, a common strategy is to delay harvest. This results in forages with high fiber concentrations and low digestibility [4], which limits intake by the mechanism of rumen fill [5]. In the Nordic countries, timothy (Phleum pratense) and meadow fescue (Festuca pratensis) are frequently used in mixed grass silages. However, their suitability in diets to mature beef cows has been questioned due to their relatively high digestibility, which may result in unnecessary large feed consumption and, consequently, excessive energy and protein intakes in relation to cow requirements [2]. 

Festulolium cultivars, i.e., hybrids between ryegrasses (Lolium spp.) and fescues (Festuca spp.), and reed canarygrass (Phalaris Arundinacea) are proposed alternatives to the traditional timothy-meadow fescue based forage. Festulolium has displayed lower in vitro digestibility of organic matter (OM) than timothy and meadow fescue when cut at the same date of the primary growth [6]. Furthermore, reed canarygrass has been shown to have greater concentrations of neutral detergent fiber (NDF) and indigestible NDF, and lower in vitro digestibility of NDF than timothy when cut at the same stage of maturity [4,7]. Intake and utilization of festulolium and reed canarygrass have been studied in dairy cows [8,9] and sheep [10], but to our knowledge, there are no studies in the literature where late cut silage of these forages have been fed to gestating beef cows. Mixing cereal straws, which have greater fiber concentration and poorer digestibility compared to grasses [11], into the silage could be one alternative to increase fiber concentration and reduce feed digestibility. However, the mean herd size of beef cows in Sweden is small, 20 cows/farm, meaning that a mixer wagon is not an economically possible investment. In addition, a large proportion of the Swedish beef cows are found in the forest districts with less favorable conditions for grain production and where access to cereal straw, hence, may be limited and/or very expensive. However, cereal straws could be economic alternatives to traditional grass forage for mature beef cows in areas in close vicinity to grain producing regions if supplemented with a source of protein. Ammoniated straw has previously been evaluated as feed for gestating beef cows [12,13]. However, ammonia-treated straw is not commonly applied in practice in our region and is not allowed in organic production. Furthermore, trials evaluating non-treated cereal straws fed together with a protein supplement to mature beef cows are scarce. 

Prepartum nutrition affects the cow’s ability to successfully give birth to a live calf and produce a heavy and live calf to weaning. Over-conditioning of beef cows prepartum is both expensive and increases the risk of dystocia [14]. On the other hand, negative energy status prepartum and poor body condition at parturition may have negative effects on cow reproductive performance [15] and on calf weight gain and weaning weight [16]. Therefore, it is important to assess the effects of new dietary regimens on cow nutritional status before these are applied in practice, in order to evaluate the eventual consequences on production output.

The objective of this study was to compare alternative roughage-based diets to a traditional timothy-meadow fescue silage, in terms of their effects on mature cow energy status prepartum and around parturition. We hypothesized that the experimental roughages would differ in digestibility and, hence, result in different planes of nutrition when fed ad libitum prepartum, which would be reflected in differences in body weight (BW) and body condition score (BCS) changes and in the metabolic profile of the periparturient cow.

## 2. Materials and Methods 

The study was conducted at Götala Beef and Lamb Research Centre, Swedish University of Agricultural Sciences, south-western Sweden, from October 2013 to October 2014. All experimental procedures were approved by the Gothenburg Research Animal Ethics Committee (case number 175-2012) and complied with the Swedish Animal Welfare Ordinance (SFS 1988:539) and Swedish Board of Agriculture regulations and general recommendations on laboratory animals (SJVFS 2012:26). 

### 2.1. Animals and Experimental Design

The study was performed with 36 spring-calving, multiparous, non-lactating beef cows of Hereford breed that were pregnant by natural mating at pasture. After a six-month grazing period the cows were housed in a free-stall barn with scraped alley and peat deep litter and fed a mixed diet of barley straw and grass silage according to requirements [3] for at least one week before the start of the experiment. Initial mean cow BW, age, and BCS (1 = emaciated, 9 = obese) [17] at experimental start was 720 ± 88 kg, 4.4 ± 1.5 years, and 5.9 ± 0.9, respectively. A randomized complete block design was used, with cows blocked into three calving groups (12 cows per group) based on expected date of calving: early (E; expected calving 18 February to 2 March), intermediate (I; expected calving 13 March to 4 April), and late (L; expected calving 12 April to 12 May). Cows within a calving group were randomly allocated to one of four pens (three cows per pen) and each pen was randomly assigned to one of four experimental roughage-based diets prepartum. After parturition, the cows were moved to new pens within 12 h, comingled with cows from other treatment groups, and all fed the same timothy silage ad libitum to meet or exceed requirements [3].

The three calving groups had different experimental start dates, i.e., when they started to receive the prepartum experimental diets. The experimental treatments were set to start approximately 112 days before the first cow in each calving group was expected to calve. As the cows calved on different days in relation to the experimental start date, cows were fed the experimental prepartum diet over a time-period from 81 to 159 days prepartum (mean 126 ± 19 d). Cows were then fed the postpartum diet for 21 to 55 days (mean 28 ± 10 d) after calving, depending on time of calving in relation to turn-out to pasture. From 24 April, cows were continuously let out on pasture 21 days after calving. Cows and calves grazed together (152 ± 15 d) on 28 ha open semi-natural pasture, dominated by tufted hair-grass (Deschampsia cespitosa). The breeding season started on 10 June and ended on 27 August. Calves were weaned on 3 October and the weaned cows continued grazing until they were housed on 29 October 2014, when the study ended.

### 2.2. Experimental Diets and Feeding

The four experimental roughage-based diets were: timothy and meadow fescue (TM) cut from the same mixed sward, festulolium (FE) cut from a monoculture sward of the festulolium hybrid Hykor of Italian ryegrass (Lolium multiflorum L.) and tall fescue (Festulolium arundinacea L.), reed canarygrass (RC; cv. Palaton) cut from a monoculture sward, and barley straw (BR; Hordeum vulgare cv. Rosalina). The grasses were harvested during primary growth from 7th to 9th July in the stage of flowering and were prewilted and preserved as round bales with addition of 2 L/t of a chemical additive (Kofasil LP; nitrite, hexamine, benzoate; Addcon Europe GmbH, Bitterfeld-Wolfen, Germany). The barley straw was baled after threshing. The postpartum diet consisted of timothy grass silage, which was harvested during primary growth on June 18th according to the procedure described above for the grass silages. 

A sufficient level of protein in the diet is necessary in order for optimal ruminal NDF digestion and microbial protein synthesis. In the NorFor digestive model, the protein balance in the rumen (PBV; g/kg dry matter; DM) is used to evaluate the adequacy of rumen protein supply for microbial growth [18]. The PBV value is calculated as: PBV = rumen degradable protein + recirculation of urea in saliva – microbial protein, where recirculation of urea is estimated to be 4.6% of the crude protein (CP) concentration in the diet [18]. The PBV value of the experimental roughages was estimated assuming a daily intake level of 8 kg dry matter (DM). The PBV value of a balanced diet should be between 10 and 40 g/kg DM [18], but for the festulolium silage and barley straw the PBV values were negative. Hence, these diets were supplemented with urea (6.9 and 11.3 g urea per kg DM, respectively) to avoid constraint of microbial CP synthesis due to lack of nitrogen (N) [19]. The urea was dissolved in water and mixed thoroughly into the festulolium silage and barley straw in a mixer wagon (Dunker TVS 120, Storti, Netherlands) before feeding and feed sampling. Cows offered the barley straw and urea mix were also fed 0.48 kg DM rapeseed meal (DM 869 g/kg, CP 378 g/kg DM, ash 69 g/kg DM, net energy (NE) 6.7 MJ/kg DM, metabolizable protein (MP) 145 g/kg DM, NDF 275 g/kg DM, PBV 22 g/kg DM, and 78.5% in vivo OM digestibility) daily from experimental start until eight weeks before the first cow in each calving group was expected to calve, in order to cover their requirements of MP [3]. Thereafter, the amount of rapeseed meal was increased to 0.63 kg DM per cow and daily basis until calving. Hence, the prepartum BR diet was composed of barley straw supplemented with urea and rapeseed meal, and the FE diet was composed of festulolium silage supplemented with urea. No additional energy or protein supplements were added to the mixed grass and reed canarygrass diets. 

Cows were fed the roughages ad libitum, allowing 10% refusals. Rapeseed meal supplemented to the BR diet was fed individually in separate feed stations (Biocontrol, CRFI, Rakkestad, Norway). Feed was delivered once daily, at approximately 0800 h, and refusals were removed twice a week. Each roughage was thoroughly mixed in the mixer wagon prior to feeding, to ensure similar particle length of each diet and with addition of urea to the FE and BR diets. Cows had free access to water, vitaminized minerals, and a salt block during the indoor period and at pasture. Calves had access to creep feeding with free access to hay during the indoor period. 

The NorFor digestive model was used to estimate the concentration of NE and MP for the roughages at a daily intake level of 8 kg of DM [20]. The concentration of metabolizable energy was calculated from the estimated apparent total tract digestible CP, crude fat, and carbohydrates [18] and NE was derived from the partial utilization efficiency (k) of metabolizable energy [21,22]. The MP concentration was estimated as the sum of undegraded dietary protein, rumen microbial protein, and endogenous protein digested and absorbed from the small intestine [18,23].

The supply relative to the requirement of NE and MP for the prepartum and postpartum periods was calculated as daily intake minus daily requirements for maintenance, gestation, and milk production [24]. Milk production in week 1, 2, and 3 postpartum was estimated to be 6, 7, and 8 kg/day, respectively, with milk fat and protein concentrations of 4.0 % and 3.4%, respectively, according to NRC [25].

The intention with rapeseed meal supplementation of the BR diet was to meet the MP requirements of the BR cows. The MP concentration of the experimental diets was estimated before initiation of the study according to the Nordic protein evaluation system for ruminants [26]. However, the NorFor digestive model was used to estimate the subsequent results of the experimental diet MP concentrations and MP intake in relation to requirements, as described above. The estimation by the NorFor digestive model revealed that cows fed the BR diet were deficient in MP prepartum. The principal difference between the system developed by Madsen [26] and the NorFor digestive model is that the latter estimates the animal’s dietary protein supply by taking into account the effects of feeding level and the total diet composition on both ration digestibility and microbial activity, and the PBV value includes estimated recirculation of urea [23].

### 2.3. Data Collection

Data on intake, BW, and BCS are reported for week -16 to -1, defined as the prepartum experimental period, and from day +1 to +21 postpartum, defined as the postpartum experimental period. Daily feed intake was recorded individually and continuously using automatic feed mangers placed on weighing cells (Biocontrol, CRFI, Rakkestad, Norway). Feed intake on the day of calving (day 0) was excluded from the intake data. The change in dry matter intake (DMI) during the last trimester was calculated by subtracting the average DMI in week -12 from the average DMI in week -1. 

Throughout the experiment, two independent technicians performed body condition scoring (1 = emaciated, 9 = obese) [17] simultaneously and a mean BCS for each cow was calculated on each occasion. All BW recordings were performed without restriction of feed and water at the same time of day after feed delivery. Cows were weighed and condition scored on two consecutive days just before the start of the experiment. Thereafter, weighing was carried out once every second week from week -16 to -9 and twice per week from week -8 to -1. Body condition score was assessed every second week from week -16 to -5 and every week from week -4 to -1. Mean BW and BCS for the weeks prepartum without recorded data were calculated as the mean of recorded values in the weeks immediately before and after those weeks. Postpartum, BW was recorded on days 1 and 2, and on days 20 and 21. On day 21, BCS was also assessed. Calves were weighed within 24 h of birth and at weaning, to measure pre-weaning calf average daily gain. Weaning weight was adjusted to 200-d weaning weight [200-d weight = birth weight + 200 × ((weaning weight−birth weight)/weaning age in days)], with no adjustment for sex of calf or age of dam. Cow BW and BCS were recorded on two consecutive days at weaning (October 2 and 3) and at the start of the subsequent indoor period in 2014 (October 28 and 29).

Changes in BW and BCS over time from week -16 prepartum until day 1 and 21 postpartum were calculated for each cow as the change per week in relation to a baseline value, i.e., the BW and BCS recorded just prior to the start of the prepartum experimental period. Changes in BW and BCS during the subsequent grazing period were calculated as the difference between the BW and BCS recorded on day 21 postpartum and the BW and BCS recorded at weaning and at the end of the grazing period. The first BW registered postpartum (BW corrected for gestation) reflects the loss of the fetus, placental membranes, and associated fluids. Seven cows calved earlier than expected and lengths of their prepartum experimental periods were thus 12 (TM, n = 1), 14 (RC, n = 1, BR, n = 1), and 15 (RC, n = 1, TM, n = 1, BR, n = 2) weeks.

### 2.4. Feed Sampling and Analysis

Representative samples of the roughages were collected daily after mixing prior to feeding. Hence, sampling of the festulolium, and of the barley straw was performed after urea was mixed into these roughages. Samples were pooled by week for weekly determination of DM content, which was used to calculate DM intake. Daily feed samples were also pooled into three-week composite samples (TM n = 8, FE n = 9, RC n = 10, BR n = 9) for later analysis of nutrient composition, which were used to calculate cow intake of NE, CP and MP over time, and to one sample per diet for analysis of pH, water soluble carbohydrates (WSCs) and fermentation products. All samples were stored frozen at −20°C for later analysis. The rapeseed meal was sampled twice during the experiment. 

Dry matter was determined in a drying cabinet at 60 °C for 24 h. Pooled roughage samples were analyzed for concentrations of OM-corrected NDF (aNDFom), OM-corrected acid detergent fiber (ADFom), and acid detergent lignin (ADL) by the FiberTech method according to van Soest [27]. The NDF analysis included α-amylase, but not sodium sulfite. The Kjeldahl procedure was used to analyze total N concentration and the concentration of CP was calculated as total N x 6.25. Crude ash concentration was determined at 525 °C for 16 h. The in vitro organic matter digestibility (IVOMD) of TM, FE, and RC feed samples was analyzed by the VOS method (organic matter digestibility of feedstuff in vitro; incubation of 0.5 g dried sample in 49 mL buffer and 1 mL rumen fluid at 38 °C for 96 h) according to Lindgren [28,29,30]. The BR and the rapeseed meal were analyzed separately for IVOMD by the EFOS method [31]. For the rapeseed meal, the aNDFom concentration was analyzed according to Chai and Udén [32], crude ash was determined after combustion at 550 °C for 6 h, and the CP concentration was determined by Dumas combustion. 

For the grass silages, lactic acid concentration was determined by high pressure liquid chromatography [33], acetate, propionate, butyrate, and ethanol by gas chromatography [34], and ammonia concentration photometrically by Scalar (CFSA, 2004) based on the Berthelot reaction. The concentration of WSC was determined according to Lengerken and Zimmermann [35] and pH was measured potentiometrically using a calibrated pH electrode.

### 2.5. Blood Sampling and Analysis

Blood (6 mL) was collected from the tail vein of the cows into evacuated tubes containing EDTA as anticoagulant (BD Vacutainer, BD-Plymouth, UK). All cows were sampled just prior to experimental start (zero sample; mean week -19 ± 2.5 prepartum). Thereafter, cows were sampled in mid-gestation, i.e., week -13 (± 2.5) prepartum, once per week from week -8 to -3, and twice per week from week -2 to -1 prepartum. Postpartum, blood was sampled within 12 h of calving (3 h ± 4.1, min 0 h, max 12 h), at 24 h (24 h ± 4.7) and 48 h (48 h ± 2.9) postpartum, and on days 6 and 10 postpartum. All blood samples were collected at approximately the same time of the day (between 1100 and 1400 h), except those related to a specific number of hours after parturition. All samples were kept on ice before being centrifuged (13 min at 1500× g) within 20 min of sampling. The plasma samples were stored at −20 °C until analysis.

All plasma samples were analyzed for concentrations of glucose, beta-hydroxybutyrate (BHBA), non-esterified fatty acids (NEFA), triglycerides, and blood urea nitrogen (BUN) as previously described [36]. Insulin was analyzed in zero samples, in samples collected in weeks -4 to -1 prepartum, and in all samples collected postpartum, using a Mercodia bovine Insulin ELISA kit (Mercodia AB, Uppsala, Sweden; intra- and inter-assay coefficient of variation < 7.5%). Samples with concentrations below the lower detection limit (lowest standard value, 0.025 µg/L) were assigned a value of 50% of the lower detection limit to allow their inclusion in statistical analyses.

### 2.6. Statistical Analysis

Two cows bearing twins (FE, n = 1 and BR, n = 1) were excluded from the analysis of data. Three cows did not have blood data for week -13 prepartum (TM, n = 1, RC, n = 1, BR, n = 1). Two cows (TM, n = 1; RC, n =1) lost their calves at parturition and the postpartum data from these cows therefore only include the BW recorded just after parturition and the first three blood samples. Two cows (TM) were diagnosed with metritis, on days 5 and 8 postpartum, respectively. Therefore, their feed intake data from days 1 to 14 postpartum and their blood data for days 6 and 10 postpartum were excluded from the analysis. Nine cow-calf pairs (TM, n = 2; FE, n = 2; RC, n = 2; BR, n = 3), were returned to their original herds after the end of the 21-d postpartum experimental period. Hence, cow and calf numbers at weaning and at the end of the grazing period on the different diets were n = 6 for TM, n = 5 for FE, n = 8 for RC, and n = 5 for BR.

Data were subjected to ANOVA with a randomized design using the mixed procedure in SAS (SAS ver. 9.3, SAS Institute, Cary, NC, USA, 2012). Cow daily DMI and blood samples collected twice per week in week -2 and -1 prepartum were reduced to weekly means for each cow before statistical analysis. The number of days that each cow was fed the experimental diet before parturition was included as a covariate in all models, but to simplify the formulas below, the covariate is not included. Data from the pre- and postpartum periods were analyzed separately. The model used to analyze data on DM, NE, CP and MP intake, NE and MP supply in relation to requirements, BW and BCS, and data on plasma metabolite and hormone concentrations in the zero and mid-gestation sample (week -13) were analyzed with the following statistical model:

*y_ij_ = µ + α_i_ + b_j_ + e_ij_* (1)

where y_ij_ is the dependent variable, µ is the overall mean, α_i_ is the fixed effect of diet (i = 1, 2, 3, 4, b_j_ is the random effect of calving group (j = 1, 2, 3) and e_ij_ is the residual error. 

Changes in BW and BCS prepartum, and changes in blood metabolite and hormone concentrations pre- and postpartum were analyzed with the statistical model:

y_ijkl_ = µ + α_i_ + b_j_ + τ_k_ + c_l(ij)_ + (ατ)_ik_ + e_ijkl_ (2)

where y_ijkl_ is the dependent variable, µ is the overall mean, α_i_ is the fixed effect of diet (i = 1, 2, 3, 4), b_j_ is the random effect of calving group (j = 1, 2, 3), *τ*_k_ is the fixed effect of time, where k = −16,…, −1 prepartum, and k = 1,…,10 postpartum, c_l(ij)_ is cow nested within calving group and diet, (α*τ*)_ik_ is the interaction between diet and time, and e_ijkl_ is the residual error. The covariance structure used for repeated factors was the AR(1) option in SAS for all parameters with evenly spaced data and the CS option for parameters with unevenly spaced data.

For analysis of calf birth weight, mean growth and weaning weight the statistical model was:

y_ijkl_ = µ + α_i_ + b_j_ + γ_k_ + θ_l_ + e_ijkl_ (3)

where y_ijkl_ is the dependent variable, α α_i_ is the fixed effect of diet (i = 1, 2, 3, 4), b_j_ is the random effect of calving group (j = 1, 2, 3), γ_k_ is the fixed effect of sex (k = 1, 2), *θ*_l_ is the fixed effect of sire (l = 1, 2, 3), and e_ijkl_ is the residual error. 

Simple linear regression analyses were performed for BW change per unit BCS change prepartum, for plasma NEFA concentration versus BCS change prepartum and for plasma BHBA concentration versus plasma NEFA concentration prepartum. For F-values, significance level was set at *p* < 0.05 and a tendency was assumed at 0.05 < *p* < 0.10. 

## 3. Results

The nutrient composition of the experimental roughages is shown in Table 1. Cows were offered the TM, FE, RC, and BR diets for 119 (± 17), 130 (± 12), 131 (± 22), and 125 (± 22) days prepartum, respectively.

### 3.1. Intake

Prepartum intakes of DM, NE, CP and MP differed among the experimental diets, as did NE and MP supply in relation to requirements (Table 2). Cows fed the TM and FE diets had greater daily DMI and greater intake in percentage of BW than cows fed the RC and BR diets. Prepartum diet tended (*p* = 0.064) to have an effect on the reduction in DMI during the last 12 weeks before parturition, where cows fed the TM, FE, RC, and BR diets decreased their intake by 2.0, 2.4, 0.67, and 0.14 kg/d, respectively, from week -12 to -1. The FE diet resulted in the highest NE intake prepartum, followed by the TM, RC, and BR diets. Cows fed the TM and FE diets had the highest intake of MP and cows fed the BR diet had the lowest intake of MP, with cows fed the RC diet being intermediate. 

Feeding the TM and FE diets prepartum resulted in intakes of NE that exceeded the recommended level [24], whereas the RC and BR diets supplied less NE than the recommended amount (Table 2). In addition, cows fed the TM and FE diets were fed MP above requirements, while MP intake was close to requirements for cows fed the RC diet and below requirements for cows fed the BR diet (Table 2). Postpartum, there was no effect of prepartum experimental diet on any of the intake variables.

### 3.2. BW and BCS

**Figure 1:**
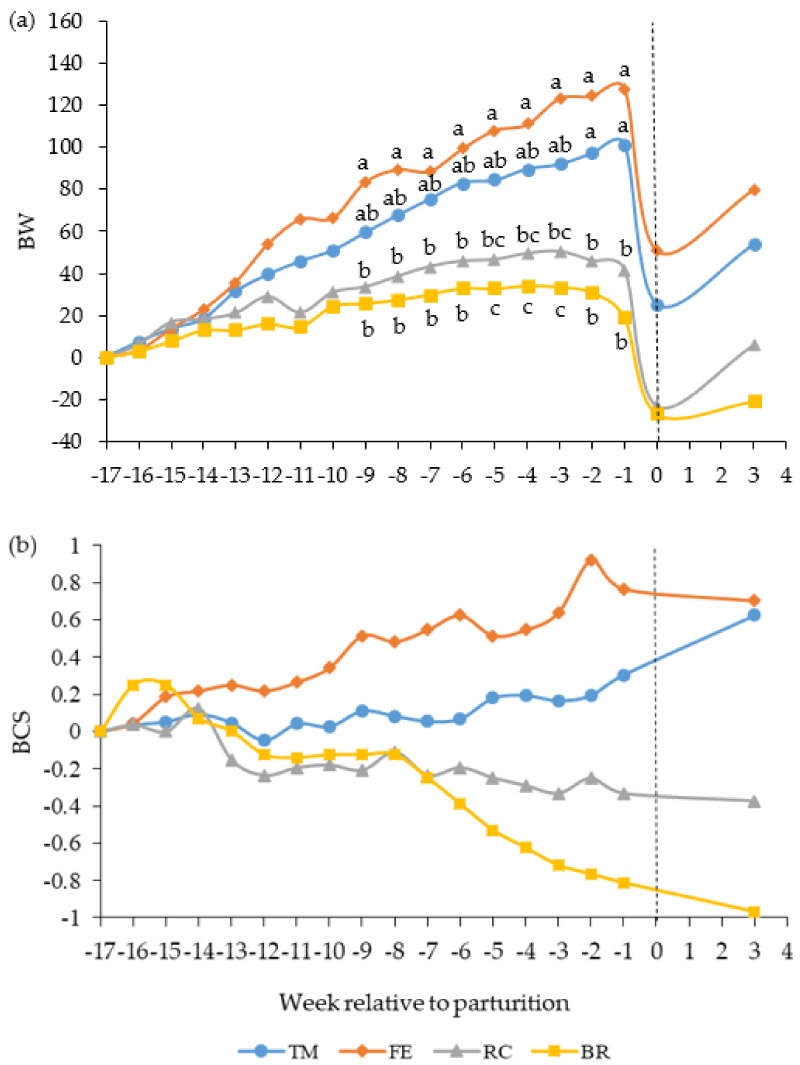
Change in (**a**) body weight (BW, kg) and (**b**) body condition score (BCS, scale 1 to 9) in beef cows fed timothy-meadow fescue silage (TM), festulolium silage plus urea mix (FE), reed canarygrass silage (RC), or barley straw plus urea mix (BR) ad libitum prepartum, including additional supplementation of rapeseed meal for cows on the BR diet. All cows received the same timothy grass silage ad libitum postpartum. Prepartum *n* = 8–9 cows/diet in each data point and postpartum n = 8 cows/diet in each data point. Time is expressed in weeks in relation to parturition (week 0) and the dashed line indicates time of calving. The interaction of diet × week was significant (*p* < 0.001) for the change in BW and tended to differ (*p* = 0.06) for the change in BCS. ^a,b,c^ items lacking a common superscript differ among weeks (*p* < 0.05).

Cows fed the FE diet increased BW gain at a faster rate than cows fed the RC and BR diets during the last nine weeks before parturition and cows fed the TM diet increased BW gain at a faster rate than cows fed the RC and BR diets during the last two and five weeks prepartum, respectively (diet × week, *p* < 0.001, Figure 1a). Averaged over the prepartum period, cows fed the TM and FE diets gained more BW than cows fed the RC and BR diets (Table 3). The BW change corrected for gestation indicated that cows fed the TM and FE diets prepartum had increased their BW by 3.3% and 6.6%, respectively, at parturition, whereas cows fed the RC and BR diets had lost 3.2% and 3.8%, respectively, of their initial BW. Cows fed the FE diet lost more BW from day 21 postpartum until the end of the grazing period than cows fed the RC and BR diets. There was no effect of prepartum diet on cow BW at weaning (3 October for all groups) or at the end of the grazing period (24 October for all groups), i.e., approximately one year after the start of the experiment. 

The interaction between diet and week for the change in BCS tended to differ prepartum (*p* = 0.06, Figure 1b). Cows fed the TM and FE diets gained body condition prepartum, whereas cows fed the RC and BR diets lost body condition (Table 3). Cows fed the FE diet had greater BCS than cows fed the BR diet one week before parturition. Cows fed the TM and FE diets lost BCS from day 21 postpartum to the end of the grazing period, whereas cows fed the RC and BR diets increased BCS.

Simple linear regression analysis of cow BW change versus BCS change prepartum indicated that one unit of BCS change corresponded to 36 kg gain/loss in BW (y = 35.81x + 6.88, r = 0.71, RMSE = 31.1, *p* < 0.001, n = 34).

### 3.3. Calf Performance

There was no effect of prepartum experimental diet on calf birth weight, adjusted 200-d weaning weight, or calf daily growth rate until weaning (Table 4).

### 3.4. Plasma Metabolite and Hormone Concentrations

**Figure 2:**
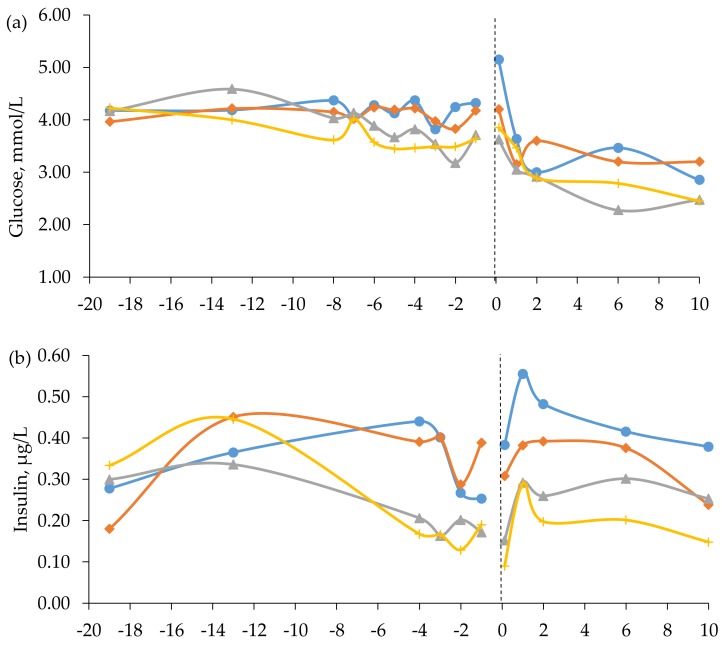

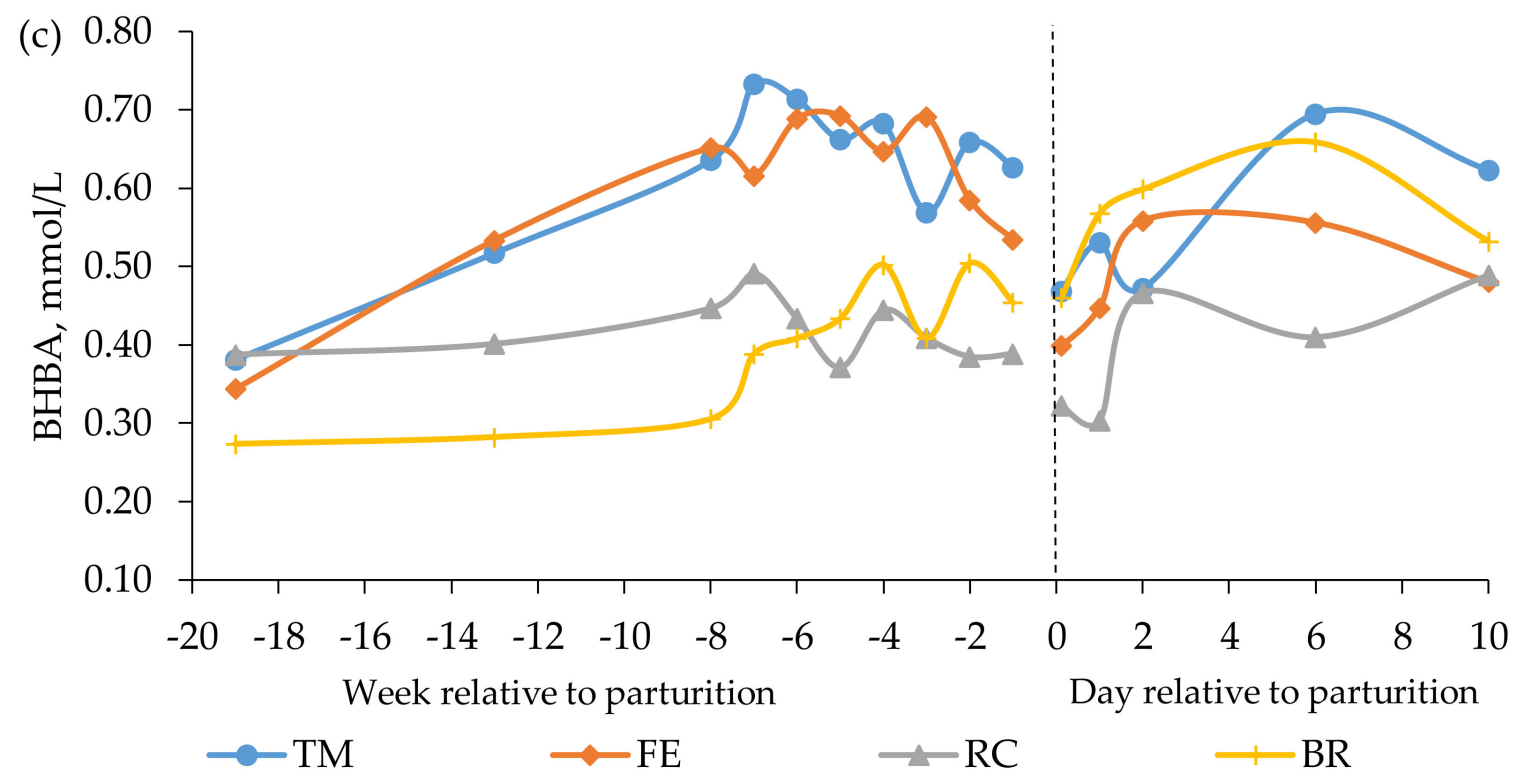
Blood concentrations of (**a**) glucose, (**b**) insulin, and (**c**) beta-hydroxybutyrate (BHBA) in beef cows fed timothy-meadow fescue silage (TM), festulolium silage plus urea mix (FE), reed canarygrass silage (RC), and barley straw plus urea mix (BR) ad libitum prepartum, including additional supplementation of rapeseed meal for cows on the BR diet. All cows received the same timothy grass silage ad libitum postpartum. Prepartum week -19 indicates the zero value (sampled prior to initiation of the prepartum dietary treatments). The dashed line indicates time of calving (week 0). Blood was sampled within 3 h (± 4.1) of parturition, at 24 h (± 4.7) and 48 h (± 2.9), and on day 6 and 10 postpartum. Prepartum and postpartum n = 7–9 cows/diet in each data point. The diet × time interaction approached significance (*p* = 0.052) for prepartum plasma levels of insulin.

**Figure 3:**
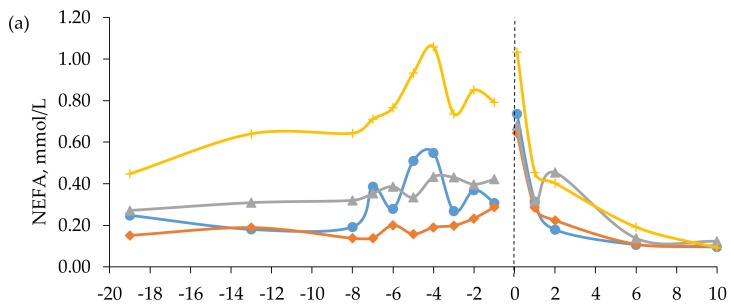

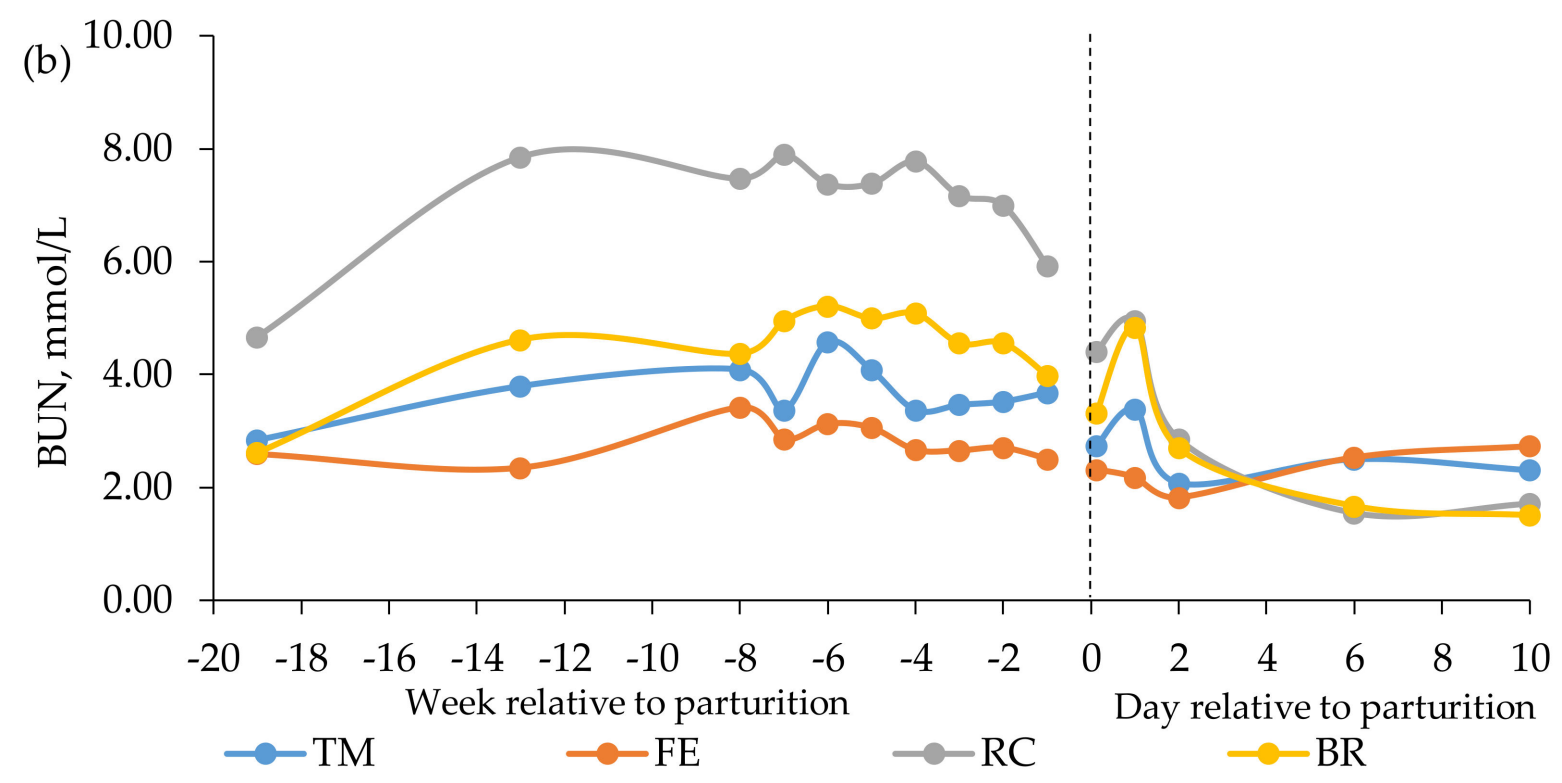
Blood concentrations of (**a**) non-esterified fatty acids (NEFA) and (**b**) blood urea nitrogen (BUN) in beef cows fed timothy-meadow fescue silage (TM), festulolium silage plus urea mix (FE), reed canarygrass silage (RC), or barley straw plus urea mix (BR) ad libitum prepartum, including additional supplementation of rapeseed meal for cows on the BR diet. All cows received the same timothy grass silage ad libitum postpartum. Week -19 prepartum indicates the zero value (sampled prior to initiation of the prepartum experimental treatments). The dashed line indicates time of calving (week 0). Blood was sampled within 3 h (± 4.1) of parturition, at 24 h (± 4.7) and 48 h (± 2.9), and on day 6 and 10 postpartum. Prepartum and postpartum n = 7 – 9 cows/diet in each data point. The interaction of diet × time was significant (*p* < 0.001) for the BUN concentration postpartum.

Results on plasma metabolite and hormone concentrations are reported in Table 5 and displayed in Figure 2 and Figure 3. Mean (± SD) blood concentration of glucose, insulin, BHBA, NEFA, triglycerides, and BUN was 4.14 ± 0.66 mmol/L, 0.27 ± 0.18 µg/L, 0.35 ± 0.12 mmol/L, 0.28 ± 0.24 mmol/L, 0.36 ± 0.08 mmol/L, and 3.2 ± 1.7 mmol/L, respectively, in the first samples before experimental start, i.e., the zero samples. The BUN concentration in the zero samples differed among diets and was higher for cows fed the RC diet than for the other cows, which had comparable levels (Table 5). The high BUN concentration could be attributed to the three RC cows in the intermediate calving group, which had BUN concentrations of 7.81 to 8.29 mmol/L in their zero samples. We have no explanation to why these three cows had higher values. If these cows were not included in the analysis, the mean BUN concentration in the zero sample was 3.12 mmol/L and there was no longer an effect of diet. 

In late gestation, i.e., week -8 to -1, cows fed the TM and FE diets had higher concentrations of glucose than cows fed the BR diet and higher concentrations of insulin and BHBA than cows fed the RC and BR diets (Table 5). The interaction between diet and time approached significance for insulin concentration prepartum (*p* = 0.052, Figure 2). When averaged across diets, glucose concentration was highest at 0 h postpartum (4.21 ± 1.48 mmol/L) and decreased thereafter, whereas the level of insulin was lowest at 0 h postpartum (0.24 ± 0.28 µg/L), followed by an increase (Figure 2). There was no effect of prepartum diet on glucose concentrations postpartum, but cows fed the TM diet had higher insulin concentrations than cows fed the BR diet (Table 5). Furthermore, cows fed the TM and BR diets had higher BHBA concentrations postpartum than cows fed the RC diet (Table 5).

In mid-gestation, i.e., week -13 (± 2.5) prepartum, plasma concentrations of NEFA were higher in cows fed the BR diet than in cows fed the TM and FE diets. In late gestation, the NEFA concentrations were higher in cows fed the BR diet than in cows fed all other diets, which had similar levels (Table 5). There was no effect of prepartum diet on plasma levels of NEFA postpartum, but when averaged across diets a peak in plasma NEFA concentration was observed at 0 h postpartum (Figure 3). Simple linear regression analysis revealed a moderate negative relationship between mean NEFA concentration and BCS change prepartum (y = −0.194x + 0.434, r = 0.53, RMSE = 0.27, *p* < 0.001, n = 34). There was no relationship between plasma concentrations of BHBA and NEFA prepartum (r = 0.0).

The higher concentration of triglycerides in week -8 to -1 in cows fed the BR diet relative to RC was attributable to one cow with approximately 100% higher triglyceride concentration at all sampling dates than the other cows fed the BR diet during this period. If this cow was excluded from the data analysis there was no effect of diet (*p* = 0.09) on prepartum concentration of this metabolite (Table 5). Prepartum diet tended to have an effect on triglyceride concentration postpartum.

Plasma concentrations of BUN in mid-gestation were higher for cows fed the RC diet than for cows fed the TM and BR diets, which, in turn, had higher BUN concentrations than cows fed the FE diet (Table 5). Feeding the RC diet resulted in the highest concentration of BUN in week -8 to -1, followed by cows fed the BR diet, with cows fed the TM and FE diets having similar and lowest levels. The interaction of diet × time was significant (*p* < 0.001) for plasma concentrations of BUN postpartum, with cows fed the RC and BR diets having higher concentrations at 24 h than cows fed the FE diet (Figure 3).

## 4. Discussion

### 4.1. Intake

Although all grass forages fed prepartum in the present study were cut within a narrow time period, i.e., 7th to 9th of July, and at similar stage of maturity, i.e., flowering, the DMI of the RC diet was lower than that of the TM and FE diets. These results are consistent with the well-documented positive relationship between forage digestibility and intake [37]. Consequently, the more digestible TM and FE diets provided NE in excess of cow requirements prepartum [24], whereas the RC diet induced a greater challenge for the cows to cover their energy demands.

A drop in daily DMI was observed during the last 12 weeks before parturition, which tended to differ among diets. Cows fed the high-energy diets, i.e., TM and FE, decreased DMI prepartum by 14 and 17%, respectively, while cows fed the RC diet decreased DMI by 8% and cows offered the BR diet maintained intake at approximately the same level. High dietary energy levels (7 MJ NE/kg DM) have previously been shown to cause more pronounced reductions in DMI before calving in dairy cows and heifers than feeding low energy diets (5 MJ NE/kg DM) [38]. Furthermore, the small decrease in daily DMI intake for cows fed the BR diet prepartum was also in agreement with the findings by Linden et al. [39], where mature beef cows fed poor-quality forage (66% NDF) did not demonstrate any reduction in intake as parturition approached. 

Kunz, et al. [40] and Douglas et al. [41] reported that dairy cows fed below NE requirements prepartum increased DMI after calving to greater levels than cows fed above NE requirements prepartum [40,41]. However, no effect of prepartum diet on postpartum intake was observed in the present study and cows managed to upregulate their feed intake to more than match their postpartum nutritional requirements [24].

### 4.2. BW and BCS

As expected, underfeeding of NE relative to requirements during gestation resulted in losses of BW and BCS, whereas overfeeding had the opposite effects [15,42]. However, care is required when drawing inferences on mobilization and deposition using BW change as an indicator, as this measure is also influenced by rumen fill [43]. Feed intake increased markedly postpartum in all cows in the present study. Thus, increased rumen fill probably explained why cows fed the RC and BR diets gained BW during the first 21 days postpartum, despite a small reduction in BCS during the same period. Cows fed the RC and BR diets gained BCS between day 21 postpartum and the end of the grazing period, although experiencing a decrease in BW during the same period. The reason for this contradictory result might also be attributed to the degree of rumen fill, as availability of pasture generally declines at the end of the growing season.

When corrected for gestation, cow BW change per unit change in BCS prepartum was 36 kg, which is similar to the 34 kg per unit of BCS reported by Buskirk et al. [44], but greater than the 30 kg per unit of BCS reported by Houghton et al. [15], when transformed to the same scale. 

During the grazing period, cows fed the RC and BR diets prepartum recovered some BCS, whereas cows fed the high-energy diets lost BCS. It was clear that these patterns of BCS change were not a response to environmental constraints, as cows were managed under the same nutritional conditions postpartum [45]. It appeared that the overfat and thin cows were ‘attempting’ to return to a target level of body fatness [46]. Similarly, Manninen et al. [47] found that beef cows, which lost BCS when fed at maintenance for 148 days prepartum, gained more BCS on subsequent pasture than their ad libitum-fed counterparts. Grazing is often a cheaper alternative to conserved roughage. Hence, a winter feeding regimen where mature cows are fed to lose some BCS prepartum and then allowed to recover on pasture is a potential strategy to reduce the annual feed costs. 

The prepartum loss of BCS in cows fed the BR diet could have affected their reproductive performance during the subsequent breeding period, as BCS at calving has been shown to influence gestation rate and length of the postpartum anestrus interval [15,42]. A BCS of 5 (scale 1 to 9) has been suggested as optimal at parturition to ensure adequate reproductive performance during the next breeding season [48,49]. Stalker et al. [16], using the same BCS scale, observed that beef cows losing BCS during gestation to calve at BCS 4.7 displayed similar reproduction results to cows calving at BCS > 5. Hence, the mean BCS of 4.9 for the BR cows in the present study might have been near the threshold at which increasing BCS no longer improves reproduction [50]. Furthermore, a higher plane of nutrition postpartum improves reproductive performance in thin cows [15,50] and all cows in our study were provided with high-quality forage during the postpartum indoor period and good pasture allowance during the grazing period. All cows were found to be pregnant again after the subsequent breeding season (results not shown) except two cows fed the FE diet. However, far-reaching conclusions about the effect of prepartum plane of nutrition on fertility rate should be made with care, as the number of cows in this study was far too limited to make such an inference.

It should be mentioned that a possible source of error in the BCS measurements could have been that the technicians were not blinded to the treatments, which could have affected their scoring of cows fed the different diets. 

### 4.3. Calf Performance

The revenue in cow-calf production is dependent on the cow weaning a heavy and healthy live calf. Thus, measures of calf performance are important when new dietary strategies prepartum are considered. Plane of nutrition prepartum may significantly alter calf birth weight [15,51,52]. However, prepartum nutritional level did not elicit differences in calf birth weight in the present study, as also experienced by others [16,49]. Furthermore, the present study found no effect of prepartum level of nutrition on calf weaning weight or weight gain until weaning, in line with previous observations [49,51]. On the contrary, Stalker et al. [16] reported lower average daily gain and weaning weight in calves born to cows losing BCS before parturition. One reason for the different results on calf performance in the studies mentioned above might be the varying degrees of over- versus undernutrition of the cows prepartum. Again, it should be noted that the number of calves within each treatment group in the present study was limited and, hence, conclusions should be drawn with caution.

### 4.4. Metabolites and Hormones

Circulating plasma glucose concentrations are generally considered to be unaffected by small dietary changes, as gluconeogenesis supplies the majority of the glucose required by ruminant tissues [53]. During undernutrition, gluconeogenesis decreases owing to the decrease in the entry of its main precursor propionate [54]. In mid-gestation, plasma glucose and insulin concentrations were similar among diets in the present study, despite relatively large differences in DMI. However, in late pregnancy when glucose demand by the growing fetus is greatly increased [53], cows fed the more digestible TM and FE diets were able to maintain stable glucose levels, whereas cows fed the BR diet experienced lower levels of glucose. Similarly, Niederecker et al. [52] reported that beef cows fed summer-baled tall fescue hay (low-energy diet) tended to have lower plasma glucose concentrations in late gestation than cows fed stockpiled tall fescue (medium-energy diet). In addition, cows fed the RC and BR diets had lower plasma levels of insulin compared to cows fed the high-energy diets. The lower DMI of cows fed the RC and BR diets and, hence, a lower absorption of glucogenic precursors, was the probable reason for this finding [41,55]. After parturition, concentrations of glucose fell to lower levels than during prepartum in all groups in the present study, which is expected when milk production initiates [38].

Postpartum, cows fed the TM diet continued to have higher plasma concentrations of insulin than cows fed the BR diet. Plasma insulin concentration is positively correlated with energy intake and propionate absorption [54]. Yet, this result could not be related to differences in glucogenic potential of the diet, as all treatment groups received the same grass silage postpartum, nor to intake levels, as DMI postpartum did not differ among groups.

Negative energy balance in ruminants results in a large release of NEFA from adipose tissue [56]. Cows fed the BR diet experienced the greatest plasma concentration of NEFA prepartum, which reflected the concomitant mobilization of fat reserves, i.e., loss of BCS, to meet the increased energy demand in late gestation [56]. This result is in agreement with Douglas et al. [41], where dry dairy cows fed NE at 80% of requirement had higher NEFA concentrations than cows fed at 160% of requirements during the last eight weeks of gestation. Although the group fed RC were apparently underfed with energy prepartum, as evidenced by the decrease in BCS, there was no difference in plasma NEFA concentrations among cows fed the TM, FE and RC diets.

Butyrate from rumen fermentation is the dominant precursor of plasma BHBA in well-fed animals, whereas in a state of negative energy balance, BHBA can also be derived partially from ketogenesis in the liver on the basis of fatty acids mobilized from adipose tissue [56,57]. In the last part of gestation of cows in the present study, the plasma BHBA concentrations were highest for the cows fed the high-energy diets. As no positive correlation could be established between plasma BHBA and NEFA concentrations, these results suggest that BHBA levels in the present study were determined primarily by rate of forestomach fermentation rather than hepatic ketogenesis.

The crude fat content of the experimental roughages was not analyzed, but grass and cereal straw are generally low in lipids. Thus, the drop in plasma triglycerides after calving was most likely related to initiation of lactation, when the mammary gland extracts triglycerides for synthesis of milk fat [58], and not to changes in the diet.

Plasma concentration of BUN has been shown to be positively related to CP intake in beef cows and heifers [59,60]. In the present study, however, the prepartum BUN levels in cows fed the RC diet were 208% and 249% higher than in cows fed the TM and FE diets, respectively, even though the TM, FE, and RC diets resulted in similar CP intakes. Plasma BUN concentration has also been shown to be strongly and positively correlated with ruminal ammonia concentration [61], which in turn can be affected by how efficiently rumen microbes can capture N in the rumen and utilize it for microbial protein synthesis. Efficient rumen microbial N use requires a balance between available N and supply of fermentable substrates, mainly carbohydrates. If energy becomes limiting, for example when poorly digestible roughages are fed, excess rumen-degradable N is degraded to ammonia and transported to the liver where it is converted to urea and later excreted in urine [62]. The higher BUN concentration in cows fed the RC diet compared with cows fed the TM and FE diets was, therefore, most likely a consequence of the lower digestibility of the RC diet. The limited amount of available fermentable substrates would also give rise to an undesired excretion of N in urine [62], which we also observed in a companion study [63], where beef cows in mid-gestation fed the RC diet had greater urinary N excretion than cows fed the TM and FE diets. Moreover, cows fed the RC diet in the present study were supplied with only 79% of their NE requirements prepartum. Thus, the elevated BUN levels in cows fed the RC diet could also partly have been a result of increased protein mobilization and associated increased amino acid oxidation to support gluconeogenesis [53]. This result is in line with the observations by Cassady et al. [64], where nutrient-restricted beef heifers had elevated plasma concentrations of BUN. Likewise, the higher BUN levels observed for cows fed the BR diet, compared with cows fed the TM and FE diets, could probably be explained by the low NE supply by the former.

## 5. Conclusions

Feeding a traditional, late cut timothy-meadow fescue-based diet ad libitum to gestating mature beef cows resulted in energy and protein intakes in excess of requirements and a concomitant increase in BW and BCS prepartum. Although all grass forages evaluated were cut within a narrow time frame at similar stages of maturity, the RC diet was less digestible, resulting in nutrient intakes that were more adapted to the nutritional demands of the gestating cow. The low nutritional value of the RC and BR diets imposed major limitations on the ability of the cows to compensate for the greater energy demand in late gestation by increasing feed intake. Hence, cows offered the RC and BR diets were in a catabolic state prepartum, as indicated by a loss of BCS and a lower plasma insulin concentration than cows fed the more digestible TM and FE diets. However, cows fed the BR diet appeared to experience a greater negative energy deficiency prepartum than cows fed the RC diet, because of the higher plasma NEFA concentrations in cows fed the BR diet. Late-cut reed canarygrass or barley straw supplemented with a proper nitrogen source may be suitable diets for mature cows in good condition prepartum, provided cows are allowed to regain lost BCS during the subsequent grazing period. Furthermore, cows fed barley straw need to be supplemented with a more digestible forage or a greater amount of protein feed as calving approaches. However, the influence of feeding NE and MP below requirements on cow reproductive performance and calf performance should be considered before making this management change.

## Figures and Tables

**Table 1:** Nutritional characteristics (mean ± SD) of the experimental roughages fed ad libitum to pregnant beef cows prepartum and of the timothy silage fed ad libitum to all cows postpartum.

Item ^2^	Prepartum ^1^				Postpartum
	TM	FE + Urea Mix	RC	BR + Urea Mix	Timothy
DM, g/kg	483 ± 29.2	361 ± 18.7	472 ± 59.9	808 ± 20.3	498 ± 73.7
Ash, g/kg DM	60.5 ± 3.81	72.0 ± 4.02	45.0 ± 2.37	59.4 ± 6.12	54.5 ± 4.42
CP, g/kg DM	111 ± 7.0	97.2 ± 3.35	130 ± 14.5	69.6 ± 5.08	128 ± 8.3
aNDFom, g/kg DM	576 ± 18.5	543 ± 13.4	648 ± 15.2	774 ± 19.2	596 ± 13.1
ADFom, g/kg DM	346 ± 9.6	319 ± 13.4	380 ± 16.0	472 ± 12.7	357 ± 10.9
ADL, g/kg DM	43.8 ± 4.70	31.3 ± 7.82	54.1 ± 5.31	59.2 ± 3.42	40.5 ± 1.71
In vitro OMD, % of OM	69.7 ± 0.67	78.6 ± 1.24	59.0 ± 2.47	37.2 ± 1.39	76.0 ± 1.94
NE, MJ/kg DM	5.8 ± 0.07	6.4 ± 0.10	5.2 ± 0.20	4.0 ± 0.07	6.5 ± 0.14
MP, g/kg DM	63 ± 0.6	63 ± 0.8	58 ± 1.6	44 ± 1.0	65 ± 1.0
PBV, g/kg DM	17 ± 6.2	−6.3 ± 7.0	45 ± 13.1	−12 ± 4.9	28 ± 8.0
WSC, g/kg DM	178	207	74	-	109
pH	4.94	4.49	4.34	-	4.55
Ethanol, g/kg DM	5.7	5.6	1.9	-	3.6
Lactic acid, g/kg DM	10.7	42.4	18.7	-	36.6
Acetic acid, g/kg DM	6.6	11.1	6.0	-	8.6
Ammonia-N, g/kg N ^3^	74	83	82	-	78

^1^ TM = timothy-meadow fescue silage, FE = festulolium silage plus urea mix, RC = reed canarygrass silage, BR = barley straw plus urea mix. Urea was added before feed sampling and analysis in amounts of 20 and 33 g CP/kg DM to the FE and BR, respectively. ^2^ CP = crude protein, aNDFom = neutral detergent fiber corrected for organic matter (OM), ADFom = acid detergent fiber corrected for OM, ADL = acid detergent lignin, OMD = OM digestibility, NE = net energy, MP = metabolizable protein, PBV = protein balance in the rumen, WSC = water-soluble carbohydrates. Values are means of n = 8 for TM and FE + urea mix, n *=* 10 for RC, n = 9 for BR + urea mix, and n = 6 for the postpartum timothy silage. For WSC and fermentation products, n = 1 for all forages. No butyric acid was detected in the silages. ^3^ Including nitrogen (N) from the nitrite and hexamine in the silage additive.

**Table 2:** Effects of prepartum and postpartum experimental diets on daily intakes of dry matter (DM), net energy (NE), crude protein (CP) and metabolizable protein (MP), and intake of NE and MP in relation to requirements [24] in gestating beef cows.

Item	Prepartum ^1^					*p*-value
	TM	FE	RC	BR	SEM	Diet
DM intake						
Prepartum, kg/d ^2^	12.9 ^a^	13.9 ^a^	9.17 ^b^	8.15 ^b^	0.413	<0.001
Postpartum, kg/d ^3^	16.5	17.5	15.7	16.7	0.844	0.52
Prepartum, % of BW	1.68 ^a^	1.64 ^a^	1.29 ^b^	1.09 ^b^	0.071	<0.001
Postpartum, % of BW	2.56	2.20	2.30	2.25	0.134	0.96
NE intake, MJ/d						
Prepartum ^2^	64.6 ^b^	80.0 ^a^	42.2 ^c^	34.6 ^d^	1.70	<0.001
Postpartum ^3^	94.4	98.4	87.9	93.1	4.73	0.47
CP intake, g/d						
Prepartum ^2^	1396 ^a^	1384 ^a^	1252 ^a^	679 ^b^	35.8	<0.001
Postpartum ^3^	2129	2209	1984	2078	130	0.65
MP intake, g/d						
Prepartum ^2^	889 ^a^	973 ^a^	513 ^b^	306 ^c^	32.7	<0.001
Postpartum ^3^	1320	1332	1210	1310	91.5	0.78
NE intake, % of requirement						
Prepartum ^2^	118 ^b^	139 ^a^	78.8 ^c^	61.0 ^d^	3.66	<0.001
Postpartum ^3^	139	137	134	142	6.72	0.86
MP intake, % of requirement						
Prepartum ^2^	166 ^b^	188 ^a^	106 ^c^	65.6 ^d^	4.16	<0.001
Postpartum ^3^	210	215	203	217	17.8	0.94

^1^ TM = timothy-meadow fescue silage, FE = festulolium silage plus urea mix, RC = reed canarygrass silage, BR = barley straw plus urea mix. All roughages were fed ad libitum prepartum and included additional rapeseed meal supplementation for cows on the BR diet. All groups received the same timothy silage ad libitum postpartum. For the TM, FE, RC, and BR diets, n = 9, 8, 9, and 8, respectively. ^2^ Mean of week -16 to -1 prepartum. ^3^ Mean of week 1 to 3 postpartum. ^a–d^ Means for the effects of diet within rows without a common superscript differ (*p* < 0.05).

**Table 3:** Effect of prepartum experimental diet on cow body weight (BW) and body condition score (BCS) in beef cows pre- and postpartum.

Item	Diet ^1^					
	TM	FE	RC	BR	SEM	*p*-value
BW, kg						
Baseline ^2,5^	714	777	694	724	27.5	0.20
At weaning ^7^	730	789	728	722	26.6	0.30
End grazing period ^7^	705	741	695	700	25.0	0.54
BW change, kg						
Prepartum ^3,5^	99 ^a^	127 ^a^	43 ^b^	22 ^b^	10.6	<0.001
Corrected for gestation ^4,5^	24 ^a^	52 ^a^	−22 ^b^	−28 ^b^	10.7	<0.001
Day 1 to 21 postpartum ^6^	35 ^a^	28 ^ab^	31 ^ab^	9 ^b^	11.8	0.03
Day 21 to weaning ^7^	−39 ^ab^	−74 ^a^	−8 ^b^	−4 ^b^	14.1	0.008
Day 21 to end of grazing period ^7^	−62 ^ab^	−120 ^a^	−42 ^b^	−26 ^b^	15.4	0.004
BCS, scale 1 to 9						
Baseline ^2,5^	6.10	5.71	5.95	5.71	0.308	0.70
Prepartum, week -1 ^5^	6.41 ^ab^	6.47 ^a^	5.61 ^ab^	4.87 ^b^	0.342	<0.001
At weaning ^7^	6.38	6.38	6.13	5.49	0.259	0.10
End grazing period ^7^	6.56	6.13	6.10	5.42	0.308	0.13
BCS change, scale 1 to 9						
Prepartum ^3,5^	0.32 ^a^	0.77 ^a^	−0.35 ^b^	−0.88 ^b^	0.359	<0.001
Week -1 prepartum to day 21 postpartum ^6^	0.37	0.08	−0.17	−0.15	0.150	0.07
Day 21 to weaning ^7^	−0.30	−0.56	0.49	0.67	0.326	0.049
Day 21 to end of grazing period ^7^	−0.36	−0.53	0.46	0.60	0.283	0.02

^1^ TM = timothy-meadow fescue silage, FE = festulolium silage plus urea mix, RC = reed canarygrass silage, BR = barley straw plus urea mix and rapeseed meal. All roughages were fed ad libitum prepartum. All groups received the same timothy silage ad libitum postpartum.; ^2^ BW and BCS recorded just prior to the start of the experimental prepartum period, which were used to calculate changes in BW and BCS.; ^3^ Prepartum change in BW and BCS = baseline value – value recorded in week -1; ^4^ BW change corrected for gestation = baseline BW – first BW recorded within 1.1 ± 0.9 days postpartum.; ^5^ n = 9, 8, 9, and 8 for the TM, FE, RC, and BR diets, respectively.; ^6^ n = 8 for all diets.; ^7^ n = 6, 5, 8, and 5 for the TM, FE, RC, and BR diets, respectively;^a-b^ Means for the effect of diet within rows without common superscripts differ (*p* < 0.05).

**Table 4:** Effect of prepartum experimental diet fed to pregnant beef cows on subsequent calf performance.

Item	Diet ^1^					
	TM	FE	RC	BR	SEM	*p*-value
Birth weight, kg ^2,4^	48.7	51.0	46.7	45.3	2.29	0.29
Weaning weight, kg ^3,5^	317	316	312	302	15.5	0.92
Growth, kg/d ^5^	1.35	1.35	1.32	1.29	0.070	0.93

^1^ TM = timothy-meadow fescue silage, FE = festulolium silage plus urea mix, RC = reed canarygrass silage, BR = barley straw plus urea mix and rapeseed meal. All roughages were fed ad libitum prepartum. All groups received the same timothy silage ad libitum postpartum. ^2^ The FE diet includes one calf with 67 kg birth weight. When this calf was removed from the data, mean birth weight of calves in the FE group was 49 kg and the effect of diet was still not significant. ^3^ Adjusted 200-d weaning weight = birth weight + 200 × [(weaning weight − birth weight)/weaning age in days], with no adjustment for sex of calf or age of the dam. ^4^ n *=* 8 for all diets. ^5^ n = 6, 5, 8, and 5 for the TM, FE, RC, and BR diets, respectively.

**Table 5:** Effect of prepartum experimental diet on pre- and postpartum plasma hormone and metabolite concentrations in beef cows.

Item ^2^		Diet ^1^					*p*-value	
		TM	FE	RC	BR	SEM	Diet	Time
Glucose,	Zero ^3^	4.15	4.04	4.18	4.19	0.324	0.94	-
mmol/L	Week -13	4.22	4.223	4.57	4.00	0.278	0.48	-
	Weeks -8 to -1	4.12^a^	4.19^a^	3.82 ^ab^	3.55 ^b^	0.313	<0.001	0.049
	Postpartum ^4^	3.50	3.49	2.98	3.09	0.247	0.12	<0.001
								
Insulin,	Zero ^3^	0.301	0.165	0.285	0.345	0.0703	0.19	-
μg/L	Week -13	0.390	0.457	0.334	0.449	0.0954	0.71	-
	Weeks -4 to -1	0.351^a^	0.369^a^	0.179 ^b^	0.166 ^b^	0.0491	<0.001	0.02
	Postpartum ^4^	0.447^a^	0.337^ab^	0.249 ^ab^	0.188 ^b^	0.0567	0.007	0.03
								
BHBA,	Zero ^3^	0.388	0.348	0.383	0.270	0.0517	0.10	-
mmol/L	Week -13	0.534 ^a^	0.541 ^a^	0.393 ^ab^	0.275 ^b^	0.0597	<0.001	-
	Weeks -8 to -1	0.663 ^a^	0.641 ^a^	0.424 ^b^	0.427 ^b^	0.0485	<0.001	0.37
	Postpartum ^4^	0.558 ^a^	0.487 ^ab^	0.393 ^b^	0.564 ^a^	0.0419	0.02	0.001
								
NEFA,	Zero ^3^	0.219	0.173	0.290	0.439	0.0800	0.07	-
mmol/L	Week -13	0.169 ^b^	0.191 ^b^	0.352 ^ab^	0.638 ^a^	0.0876	0.001	-
	Weeks -8 to -1	0.355 ^b^	0.194 ^b^	0.385 ^b^	0.821 ^a^	0.0726	<0.001	0.20
	Postpartum^4^	0.260	0.264	0.355	0.442	0.0955	0.25	<0.001
								
Trigl.,	Zero ^3^	0.335	0.369	0.349	0.403	0.0324	0.34	-
mmol/L	Week -13	0.283 ^b^	0.322 ^ab^	0.385 ^ab^	0.425 ^a^	0.0340	0.01	-
	Weeks -8 to -1	0.335 ^ab^	0.382 ^ab^	0.325 ^b^	0.417 ^a^	0.0321	0.02	<0.05
	Postpartum^4^	0.115	0.163	0.121	0.095	0.0176	0.06	0.12
								
BUN,	Zero ^3^	2.76 ^b^	2.77 ^b^	4.71 ^a^	2.55 ^b^	0.716	0.002	-
mmol/L	Week -13	4.03 ^b^	2.45 ^c^	7.74 ^a^	4.51 ^b^	0.682	<0.001	-
	Weeks -8 to -1	3.57 ^c^	2.99 ^c^	7.44 ^a^	4.67 ^b^	0.303	<0.001	0.04
	Postpartum ^4^	2.50	2.37	3.21	2.78	0.248	0.08	<0.001

^1^ TM = timothy-meadow fescue silage, FE = festulolium silage plus urea mix, RC = reed canarygrass silage, BR = barley straw plus urea mix and rapeseed meal. All roughages were fed ad libitum. All groups received the same timothy silage ad libitum postpartum. ^2^ BHBA = beta-hydroxybutyrate, NEFA = non-esterified fatty acids, Trigl. = triglycerides, BUN = blood urea N. For prepartum values, n = 9, 8, 9, and 8 for the TM, FE, RC, and BR diets, respectively. For postpartum values, n = 8 for all diets. ^3^ Zero = the blood sample collected just prior to the cows started being fed the experimental diets. ^4^ Blood was sampled within 3 h (± 4.1) of parturition, at 24 h (± 4.7) and 48 h (± 2.9), and on day 6 and 10 postpartum. ^a–c^ Means for the effect of diet within rows with different superscripts are significantly different (*p* < 0.05).
